# The sniffing bead system, an olfactory dysfunction screening tool for geriatric subjects: a cross-sectional study

**DOI:** 10.1186/s12877-020-01871-7

**Published:** 2021-01-14

**Authors:** Hyun Jin Min, Sun Mi Kim, Doug Hyun Han, Kyung Soo Kim

**Affiliations:** 1 Department of Otorhinolaryngology-Head and Neck Surgery, Chung-Ang University College of Medicine, Seoul, South Korea; 2 Department of Psychiatry, Chung-Ang University College of Medicine, Seoul, South Korea

**Keywords:** Geriatric assessment, Olfactory dysfunction, Olfactory perception, Sniffing bead system

## Abstract

**Background:**

This study aimed to develop a simple and one-off olfactory screening test, the sniffing bead system, for general clinical use in older adults**.**

**Methods:**

In this cross-sectional study, geriatric subjects (aged > 50 years) who underwent neurocognitive and olfactory function tests were included. Overall, 137 subjects were enrolled, and the study was conducted at Chung-Ang University, Seoul, Korea. Olfactory function was measured by obtaining the scores of the sniffing bead system using 2-phenylethyl alcohol, n-butanol, and the YSK olfactory function test. Time taken for each olfactory function test was also measured.

**Results:**

The score of the 2-phenylethyl alcohol sniffing bead test was 2.58 ± 1.52, which was significantly associated with the YSK_threshold (2.41 ± 1.79) (*p* < 0.001, Pearson’s correlation coefficient = 0.429), YSK_identification (8.93 ± 3.25) (*p* = 0.014, Pearson’s correlation coefficient = 0.208) and YSK_threshold-discrimination-identification (17.46 ± 5.49) (*p* < 0.001, Pearson’s correlation coefficient = 0.316) test scores. In the normal cognitive function group, YSK_threshold *(p* < 0.001, Pearson’s correlation coefficient = 0.479*)*, YSK_identification *(p* = 0.003, Pearson’s correlation coefficient = 0.316*),* and YSK_threshold-discrimination-identification (*p* < 0.001, Pearson’s correlation coefficient = 0.429) were significantly correlated with the scores of the 2-phenylethyl alcohol sniffing bead system. In the impaired cognitive function group, the YSK_threshold (*p* = 0.002, Pearson’s correlation coefficient = 0.415*)* and YSK_ threshold-discrimination-identification (*p* = 0.004, Pearson’s correlation coefficient = 0.385*)* were significantly correlated with the scores of the 2-phenylethyl alcohol sniffing bead system. Time taken for the 2-phenylethyl alcohol sniffing bead system was 5.00 ± 1.51 min, which was significantly lower than that for the YSK_threshold-discrimination-identification (20.43 ± 5.29 min) (*p* < 0.001). The scores of the 2-phenylethyl alcohol sniffing bead system were significantly correlated with those of the n-butanol sniffing bead system (3.50 ± 1.21) (*p* < 0.001, Pearson’s correlation coefficient = 0.315).

**Conclusions:**

This sniffing bead system was specifically designed for screening olfactory function in older adults, and it may allow for the rapid and accurate assessment of olfactory dysfunction.

**Supplementary Information:**

The online version contains supplementary material available at 10.1186/s12877-020-01871-7.

## Background

Olfactory dysfunction may occur during the natural aging process, and its overall prevalence is estimated at approximately 5% in the general population [1]. In the geriatric population, olfactory dysfunction affects critical functions such as nutrition, immunity, mood, and behaviour [2, 3]. More recently, olfactory dysfunction has been shown to be significantly associated with multiple neurodegenerative disorders. The pathology and development of Alzheimer’s disease (AD) and Parkinson’s disease (PD) are associated with olfactory impairment [4], and importantly, olfactory dysfunction occurs during the prodromal stage of these neurodegenerative diseases. Therefore, screening for olfactory dysfunction may be a key factor in early diagnosis in the early stages of AD [5]. Similarly, olfactory function can be deteriorated in drug-naïve PD subjects with mild cognitive impairment [6]. With the increasing prevalence of neurodegenerative diseases, their social impact is also growing, resulting in the need for olfactory dysfunction screening not only in ear-nose throat (ENT) clinics, but also in general clinical settings, especially when treating geriatric subjects.

Various olfactory tests have been developed, such as the Connecticut Chemosensory Clinical Research Center test, the University of Pennsylvania Smell Identification Test, and the Korean Version of the Sniffin’ Sticks (KVSS II) test. Although these tests are useful in evaluating olfactory function, they each require more than 20 min to complete. These tests’ durations, as well as their complexity, make them tedious for use as screening tests [7]. Furthermore, for geriatric subjects, especially those suspected to have impaired cognitive function, the relatively long test duration is a barrier to patient compliance. Therefore, there is a need for an accurate, simple, and rapid test to screen for olfactory function in geriatric subjects.

Oh et al. reported that the first detectable n-butanol concentration, a measure obtained at the beginning of the threshold detection test during conventional olfactory testing, was significantly correlated with the overall result of the full olfactory test battery [8]. A significant design limitation of current olfactory test kits is the use of odor-impregnated felt-tip pens, which lose their strength over time due to repeated testing, thus limiting their reliability. Additionally, these felt-tip pens are not available for purchase individually, and the total cost of the testing kit can be prohibitively expensive. Therefore, we aimed to develop a simple, easy-to-use screening tool for olfactory dysfunction. We developed the sniffing bead system, which makes use of a uniquely designed odor-packed bead that is directly released at the nostrils of the subject and enables simple and easy screening for olfactory function in various clinical settings.

## Methods

### Participant recruitment

Patients who visited the neuropsychiatric department of our institute with a pre-existing mild cognitive impairment (MCI) or dementia diagnosis, individuals on their first visit for cognitive assessment, and individuals who responded to a hospital bulletin advertisement were enrolled. Patients who agreed to undergo both cognitive function testing and olfactory function testing between August 2018 and May 2019 were enrolled in this study. The inclusion criteria were (a) age > 50 years, and (b) being capable of complying with the study protocol. On the other hand, the exclusion criteria were as follows: (a) any past or current diagnosis of brain tumor, epilepsy, PD, major depressive disorder, bipolar disorder, or schizophrenia disease; (b) history of head trauma or stroke; (c) diagnosis of allergic rhinitis, acute/chronic rhinosinusitis, or a history of nasal surgery; (d) communication difficulties from severe hearing impairment or aphasia; and (e) failure to understand the study protocol and objectives. Information about the presence of the exclusion criteria was obtained through self-reporting by the enrolled subjects. Data on the demographic and clinical characteristics, including age, sex, smoking status, and diagnosis of AD or vascular dementia, were obtained from all the subjects. All experimental protocols were approved by the Institutional Review Board of the Chung-Ang University Hospital, and written informed consent was obtained from each participant and their caregiver (e.g., spouse or adult child).

### Development of the sniffing bead system

The sniffing bead system was composed of several small beads (7 mm in diameter) and a handpiece (Additional file 1: Figure S1, Additional file 2: Video clip 1). The beads were designed such that any odor can be used. The beads containing the odorants are prepared beforehand, and the smell of the odorants can be maintained for more than 6 months if the beads are sealed. All the beads are only for a single-time use. In this study, we prepared beads packed with 2-phenylethyl alcohol (PEA), and beads with distilled water served as negative controls.


**Additional file 2:** **Video clip 1.** Video clip showing the working of the sniff bead system.

For the PEA beads, which have a rose-like smell widely used in olfactory testing [9], eight beads corresponding to eight different concentrations of PEA were used. PEA concentration was diluted in distilled water at a ratio of 1:2 (from 10 to 0.078%). The lowest concentration at which the subject detected the PEA odorant was scored as the PEA threshold score (scores: 1 [highest concentration] to 8 [lowest concentration], and 0 [not detected]).

To evaluate the generalizability of PEA sniffing bead system, n-butanol beads were prepared. For the n-butanol beads, which have an odor also commonly used for olfactory testing in the Korean population [8], eight beads corresponding to eight different concentrations of n-butanol were used. The n-butanol was diluted in distilled water at a ratio of 1:2 (from 4 to 0.0312%), with distilled water used as a negative control. The lowest concentration at which the subjects detected the n-butanol odorant was scored as the n-butanol threshold score (scores: 1 [highest concentration] to 8 [lowest concentration], and 0 [not detected]). The handpiece was composed of two parts: a plastic capsule and an aluminum body. The capsule could release the odor after bead insertion.

### Olfactory threshold testing using the sniffing bead system

All olfactory function testing procedures were performed by two trained technicians in a well-ventilated laboratory lacking other scents, odors, or air currents. At the start of the test, the technician inserted only the bead containing distilled water into the handpiece and burst the bead 2 cm from the front of both nostrils. The patient was then asked if he/she recognized its odor. If the patient answered that he/she did not recognize any scent, the bead containing the fourth-strongest concentration (0.248% for PEA; 0.499% for n-butanol) was used. If the patient still did not recognize the scent, a higher concentration bead was used. Conversely, if the patient recognized the scent, a lower concentration bead was used. The lowest concentration at which the patient recognized the scent was recorded as the threshold for the PEA sniffing bead system. The time taken for each test was measured from the beginning to the end of the test by the technician.

### YSK olfactory function testing

The YSK olfactory function test (RHICO Medical Co., Seoul, Korea) [10] is an olfactory testing instrument designed specifically with odors that are familiar to Koreans. It was used as the comparative standard olfactory function test in this study. The test battery included three subsets (i.e., threshold, discrimination, and identification tests). The test was performed in the same manner as the KVSS II [11]. In brief, the detection threshold was defined as the concentration at which the PEA (highest concentration: 10%, 1:2 serial dilutions to 12 steps) was correctly identified four times in a row. The test was performed using a triple-forced-choice paradigm and a seven-reversal initially ascending single-staircase procedure as described by Doty et al. [12]. For the discrimination test, triplets of odorants—two identical, one different—were presented, and subjects were asked to choose the odd, i.e., different odorant. Twelve pen triplets were presented to the subjects. The identification test involved a multiple forced choice from four descriptors. The sum of the three test scores was calculated as the threshold-discrimination-identification (TDI) score. The score of each test ranged from 1 to 12 for the threshold test, 0 to 12 for the discrimination test, and 0 to 12 for the identification test. The combined TDI score ranged from 1 to 36. Olfactory functions of the enrolled subjects were grouped as normosmia, hyposmia, and anosmia based on TDI score of the YSK test. The time taken for each test was measured from the beginning to the end of the test.

### Neurocognitive testing

Two psychiatrists evaluated all the subjects for cognitive function and major neurocognitive disorders using the Structured Clinical Interview for DSM-5 Disorders—Clinician version (SCID-5-CV) [13]. The Korean version of the Consortium to Establish a Registry for Alzheimer’s Disease-Korean version assessment packet (CERAD-K) [14] and the Korean version of the Mini-Mental State Examination [15] were also administered to all the subjects. The CERAD-K consists of the following items: Verbal Fluency, Modified Boston Naming, Mini-Mental State Examination, Word List Memory, Constructional Praxis, Word List Recall, Word List Recognition, Constructional Praxis Recall, Trail Making Test, and Stroop Test. We obtained each participant’s sub-scores, total score (from 0 to 100), and three cognitive function levels after adjusting for age and sex (normal range, significant decline but not dementia level, or impaired performance comparable to dementia). The subjects were divided into two groups based on the results of the CERAD-K examinations: subjects with normal cognitive function and those with impaired cognitive function [14, 16].

### Statistical analyses

All the statistical analyses were performed using SPSS version 19.0 (IBM Corp., Armonk, NY, USA). Descriptive data were presented as means ± standard deviations and categorical data as frequencies (n) and percentages (%). The Pearson’s correlation coefficient was used to evaluate the association between two variables of interest. Differences between two groups were evaluated using the independent t-test. To test for differences among the three groups, a one-way Analysis of variance (ANOVA) was used with a Bonferroni post-hoc test. A *p*-value < 0.05 was considered statistically significant.

## Results

The demographic and clinical characteristics of the subjects are summarized in Table 1. Of the 137 subjects, 28 (20.4%) were men and 109 (79.6%) were women, with the mean age being 71.60 ± 7.56 years. The mean combined TDI score, based on the YSK olfactory function test, was 17.46 ± 5.49. The mean score of the PEA and n-butanol sniffing bead systems butanol are 2.58 ± 1.52 and 3.07 ± 1.50.

**Table 1:** Characteristics of the enrolled subjects

Variables	Number
Number	137
Sex	
Male: female	28:109
Age (years)	
Mean ± SD	71.60 ± 7.56
Range	58–92
Smoking	
Non-smoker, n (%)	134 (98.5%)
Smoker, n (%)	3 (1.5%)
Number of subjects with AD (%)	38 (28.1%)
Number of subjects with VD (%)	7 (5.2%)
MMSE-K	22.08 ± 6.56
CERAD-K	60.05 ± 22.72
YSK_threshold	2.41 ± 1.79
YSK_discrimination	6.11 ± 2.07
YSK_identification	8.93 ± 3.25
YSK_TDI	17.46 ± 5.49
Mean score of PEA sniffing bead system	2.58 ± 1.52
Mean score of n-butanol sniffing bead system	3.07 ± 1.50

*AD* Alzheimer’s disease, *CERAD-K* Consortium to Establish a Registry for Alzheimer’s Disease-Korean version, *MMSE-K* Mini-Mental State Examination-Korean version, *PEA* 2-phenylethyl alcohol, *SD* Standard deviation, *VD* Vascular dementia, *YSK_TDI* YSK_threshold-discrimination-identification

**Fig. 1:**
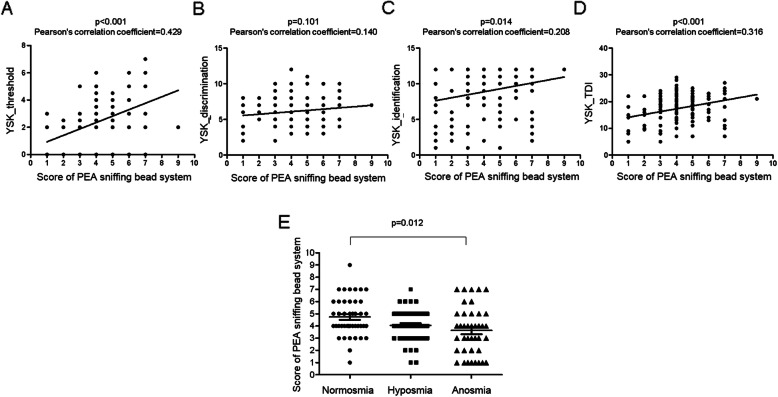
Correlation between the PEA sniffing bead system scores and the YSK olfactory function test scores. **a** Correlation between the PEA sniffing bead system and the YSK_threshold scores. **b** Correlation between the PEA sniffing bead system and the YSK_discrimination scores. **c** Correlation between the PEA sniffing bead system and the YSK_identification scores. **d** Correlation between the PEA sniffing bead system and the YSK_TDI scores. **e** Comparison of the PEA sniffing bead system scores between subjects with normosmia, hyposmia, and anosmia. PEA, 2-phenylethyl alcohol; TDI threshold-discrimination-identification

The correlation between the results of YSK olfactory function test, i.e. standard testing, and the scores of PEA sniffing bead system was evaluated. The threshold and identification scores of the YSK olfactory function test were positively correlated (YSK_threshold: *p* < 0.001, Pearson’s correlation coefficient = 0.429 and YSK_identification: *p* = 0.014, Pearson’s correlation coefficient = 0.208, respectively) with the scores of PEA sniffing bead system. However, the discrimination component of the YSK olfactory function test was not significantly correlated with the PEA sniffing bead test scores (YSK_discrimination: *p* = 0.101, Pearson’s correlation coefficient = 0.140) (Fig. 1a–c). The combined TDI score of the YSK olfactory function test was also positively correlated with the scores of PEA sniffing bead system (YSK_TDI: *p* < 0.001, Pearson’s correlation coefficient = 0.316) (Fig. 1d). When the scores of PEA sniffing bead system were compared according to olfactory function, the mean score was 4.74 ± 1.59 in subjects with normosmia, 4.05 ± 1.25 in subjects with hyposmia, and 3.62 ± 1.94 in subjects with anosmia. The difference between the subjects with normosmia and anosmia was statistically significant (*p* = 0.012) (Fig. 1e).

Subsequently, subjects were divided into groups based on cognitive function. Eighty-four subjects were classified as having normal cognitive function and 53 were classified as having impaired cognitive function. In the normal cognitive function group, the YSK_threshold (*p* < 0.001, Pearson’s correlation coefficient = 0.479), YSK_identification (*p* = 0.003, Pearson’s correlation coefficient = 0.316), and YSK_TDI (*p* < 0.001, Pearson’s correlation coefficient = 0.429) values were significantly correlated with the scores of the PEA sniffing bead system. In the impaired cognitive function group, the YSK_threshold (*p* = 0.002, Pearson’s correlation coefficient = 0.415) and YSK_TDI (*p* = 0.004, Pearson’s correlation coefficient = 0.385) values were significantly correlated with the scores of the PEA sniffing bead system (Table 2, Supplementary figure 2). The scores of the PEA sniffing bead system were not significantly different between the subjects with normal (3.98 ± 1.43) and decreased cognitive function (4.39 ± 1.89) (*p* = 0.336) (Supplementary figure 3).

**Table 2:** Correlation between each component of the olfactory function test (YSK) and the scores of the PEA sniffing bead system in subjects with and without cognitive impairment

	Normal		Cognitive impairment		Total	
	Coefficient	p	Coefficient	p	Coefficient	p
YSK_threshold	0.479	< 0.001	0.415	0.002	0.429	< 0.001
YSK_discrimination	0.195	0.074	0.185	0.183	0.140	0.101
YSK_identification	0.316	0.003	0.257	0.068	0.208	0.014
YSK_TDI	0.429	< 0.001	0.385	0.004	0.316	< 0.001

Coefficient, Pearson’s correlation coefficient; *YSK_TDI* YSK_threshold-discrimination-identification

**Fig. 2:**
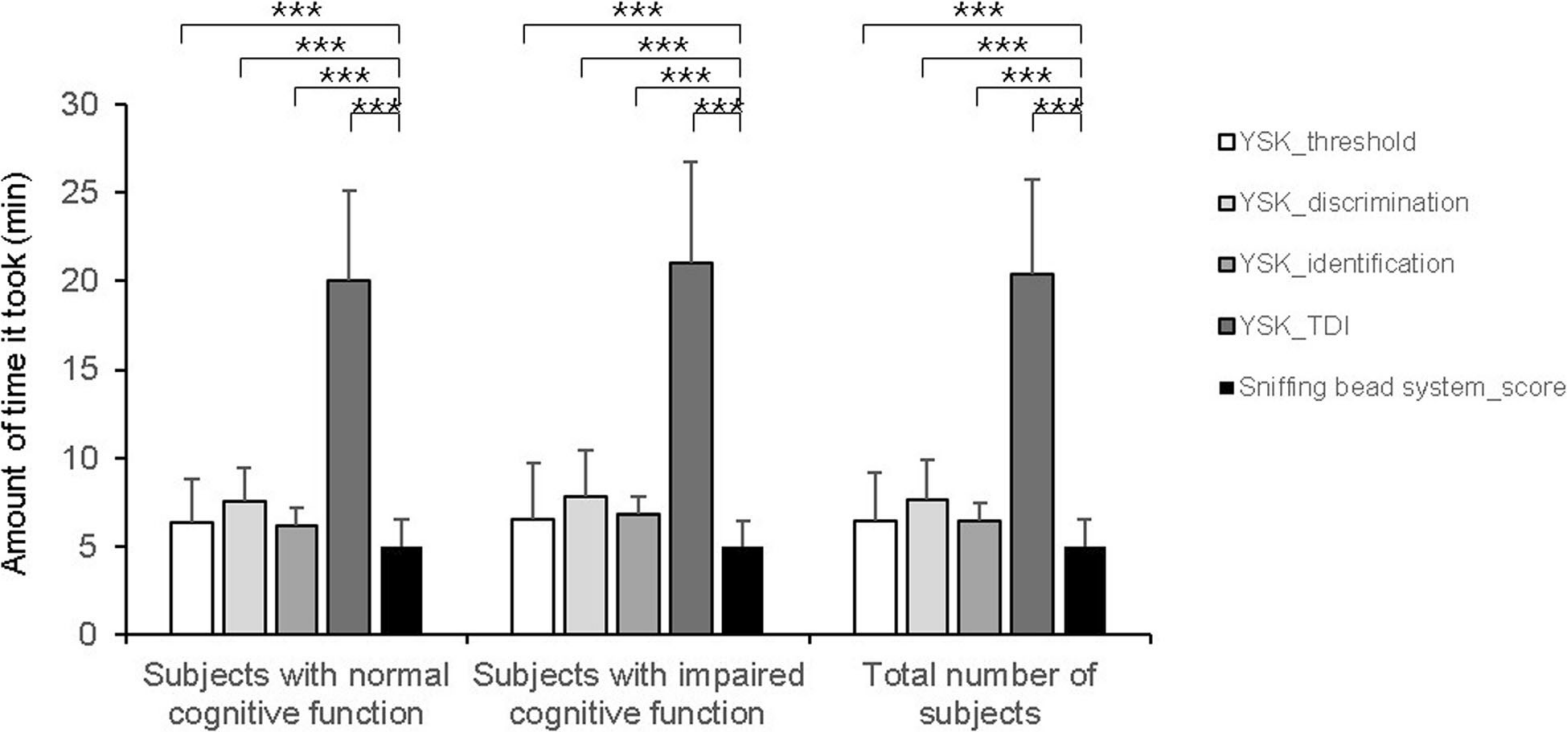
Duration of each olfactory test in subjects with normal and impaired cognitive function groups, and for the total number of subjects. *** *p* < 0.001

Next, we evaluated the time required to conduct each olfactory test. The mean duration of the YSK_threshold test was 6.41 ± 2.71 min for all subjects, with a mean test duration of 6.35 ± 2.40 min and 6.51 ± 3.17 min in subjects with normal and impaired cognitive function, respectively. The mean duration of the YSK_discrimination test was 7.62 ± 2.23 min for all the subjects, with a mean test duration of 7.50 ± 1.96 min and 7.83 ± 2.61 min in subjects with normal and impaired cognitive function, respectively. The mean duration of the YSK_identification test was 6.43 ± 1.96 min for all the subjects, with a mean test duration of 6.20 ± 1.65 min and 6.79 ± 2.35 min in subjects with normal and impaired cognitive function, respectively. The mean duration of the complete YSK olfactory function test battery was 20.43 ± 5.29 min in all the subjects, with a mean test duration of 20.05 ± 5.01 min and 21.01 ± 5.72 min in those with normal and impaired cognitive function, respectively. In contrast, the mean test duration of the PEA sniffing bead system was 5.00 ± 1.51 min for all subjects, with a mean test duration of 4.87 ± 1.36 and 5.01 ± 1.44 min in the normal and impaired cognitive functioning groups, respectively. The mean test duration of the PEA sniffing bead system (5.00 ± 1.51 min) was significantly shorter than that of the YSK_threshold (6.41 ± 2.71 min), YSK_discrimination (7.62 ± 2.23 min), YSK_identification (6.43 ± 1.96 min), and YSK_TDI tests (20.43 ± 5.29 min) (Fig. 2).

**Fig. 3:**
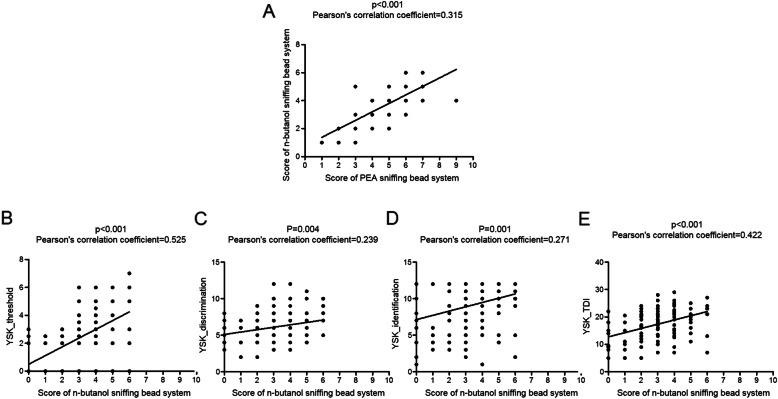
**a** Correlation between the PEA and n-butanol sniffing bead system scores. **b**-**e** Correlation between the n-butanol sniffing bead system and the YSK olfactory function test scores. **b** Correlation between the n-butanol sniffing bead system and the YSK_threshold scores. **c** Correlation between the n-butanol sniffing bead system and the YSK_discrimination scores. **d** Correlation between the n-butanol sniffing bead system and the YSK_identification scores. **e** Correlation between the n-butanol sniffing bead system and the YSK_TDI scores. PEA, 2-phenylethyl alcohol; TDI, threshold-discrimination-identification

Finally, the generalizability of the sniffing bead system to other odors was evaluated. The scores of the PEA and n-butanol sniffing bead systems were significantly correlated (*p* < 0.001, Pearson’s correlation coefficient = 0.315) (Fig. 3a). The scores of the n-butanol sniffing bead system were significantly correlated with YSK_threshold scores (*p* < 0.001, Pearson’s correlation coefficient = 0.525), YSK_discrimination (*p* = 0.004, Pearson’s correlation coefficient = 0.239), YSK_identification (*p* = 0.001, Pearson’s correlation coefficient = 0.271), and YSK_TDI (*p* < 0.001, Pearson’s correlation coefficient = 0.422) (Fig. 3b–e).

## Discussion

### Summary

The development and validation of a novel screening tool for olfactory dysfunction that specifically developed for older adults was reported in this study. The mean total score of the conventional olfactory test (YSK, which was composed of three subsets of threshold, discrimination, and identification) was 17.46 ± 5.49. The mean total score of YSK was significantly correlated with the scores of the PEA sniffing bead system in subjects with both normal and impaired cognitive function. Regarding each subset of the conventional olfactory test, the scores of the PEA sniffing bead system was significantly associated with the threshold and identification scores in the normal cognitive function group, while only the threshold score was associated with the scores of the PEA sniffing bead system in the impaired cognitive function group. When we grouped subjects into normosmia, hyposmia, and anosmia based on total score of YSK, the score of PEA sniffing bead system was significantly different between groups.

### Comparison with the existing literature

If the ability to discriminate or identify an odor is diminished, an impairment of the central nervous system may be suspected, whereas isolated threshold deficits may suggest peripheral damage [17]. A marked decrease in the olfactory identification scores was apparent in subjects with dementia, and a loss of odor identification had been proposed as a predictive marker of the transition from mild cognitive impairment to AD [9]. As PEA sniffing bead system is based on odor recognition threshold, it is possible that the score of PEA sniffing bead system does not directly accord with the identification score. The correlation between scores of the PEA sniffing bead system and identification scores, as seen in subjects with normal cognitive function, was not found in subjects with impaired cognitive function. In subjects with impaired cognitive function, who particularly has declined odor identification (among three subsets (threshold, detection, and identification) of olfactory function), the reliability of PEA sniffing bead system could be decreased.

A marked benefit of this novel olfactory testing system was the ability to use different odors within the same system. Odor identification tests have cultural biases, and the current conventional olfactory tests are not applicable across all ages and cultures [18, 19]. Modification of an odor is necessary for a test to be of use cross-culturally. The results of our sniffing bead system using both n-butanol bead and PEA as odors were compared and it was found that the results of the two odors were significantly correlated. Thus, the system can be applied using other novel odors that are culturally appropriate.

To date, several olfactory tests have been developed specifically for use in Korea. The KVSS and the YSK olfactory function tests are both composed of three subsets, and the summation of the total threshold, discrimination, and identification scores is used in ENT clinics. In this study, the focus was on geriatric subjects who required olfactory screening in diverse clinical settings, including neurology, internal medicine, and family medical clinics. These settings usually involve numerous geriatric subjects with potential olfactory dysfunction. In these clinics, performing a conventional olfactory function test is difficult and time-consuming, and thus, the diagnosis of olfactory dysfunction is often missed. Using the present olfactory function screening system, more subjects suspected to have olfactory dysfunction can be potentially referred to an ENT clinic for further evaluation.

### Implications for research and/or clinical practice

For geriatric subjects, especially those with cognitive impairment, conducting conventional olfactory testing lasting more than 20 min can be challenging. The long test duration is one of the primary reasons for poor compliance to olfactory test instructions in this patient population. Hence, the brief and easy-to-perform olfactory bead screening system could be highly beneficial as a screening tool. Although a shorter test time is critical for screening tools, it compromises on the benefits that longer, conventional olfactory testing can offer, such as distinguishing between levels of less-than-total olfactory dysfunction or the identification of malingering. Hence, such screening tools should not be used as substitutes for more extensive conventional testing.

### Study strengths and limitations

The sniffing bead system, our newly developed diagnostic system for the screening of olfactory function, is simple, easy-to-perform, not culturally specific, and particularly suited for testing older adults. It is based on single-use beads containing odorous solutions and overcomes the primary disadvantage of conventional, felt-tip-pen-type olfactory test kits that degrade over time.

In geriatric subjects, deficits in olfaction are difficult to detect unless they are specifically assessed, especially in those with impaired cognitive function. Based on this validation study, the sniffing bead system is a useful screening tool that quantifies detection thresholds comparable to those in conventional olfactory testing, but in a significantly shorter time. The validity of this system is further supported in cognitively impaired geriatric subjects, including subjects with AD and vascular dementia.

However, several limitations of the study should be considered. First, most of the subjects were women. Men have been reported to generally have poorer olfactory function than women [20]; however, another study failed to find a correlation between sex and odor identification [21]. Given this inconsistency in the literature, there is a need to compare the results of the sniffing bead system between men and women. Second, the relatively small sample size and restricted ethnicity limit the generalizability of the results. As the sniffing bead system can be adapted to other odors, a large population-based study including participants of various ethnicities is needed. Finally, this study only evaluated the usefulness of this olfactory screening tool but did not evaluate the threshold of the sniffing bead system for diagnosis of diseases such as AD, as has been done for previous conventional olfactory function tests. Further studies investigating the threshold for the diagnosis of diseases should be performed.

## Conclusions

The development and validation of a novel screening tool for olfactory dysfunction, specifically for use in older adults, has been reported in this study. The sniffing bead system may be applied both as a screening tool for olfactory dysfunction and for follow-up of normal olfactory function in the geriatric population. As it is a simple, quick, and one-off system, it can be widely applied in various clinical fields.

## Supplementary Information

**Additional file 1:** **Figure S1.** Picture of sniffing bead system.

**Additional file 3:** **Figure S2.** Correlation between each component of the olfactory function test (YSK) and the PEA sniffing bead system scores in subjects with normal cognitive function (A–D) and impaired cognitive function (E–H).

**Additional file 4:** **Figure S3.** Comparison of the PEA sniffing bead system scores between subjects with normal and decreased cognitive function.

## Data Availability

The datasets generated and/or analyzed during the current study are available from the corresponding author on reasonable request.

## Abbreviations

AD: Alzheimer’s disease
CERAD-K: Consortium to Establish a Registry for Alzheimer’s Disease-Korean version assessment packet
KVSS II: Korean Version of Sniffin’ Sticks
PD: Parkinson’s disease
PEA: 2-phenylethyl alcohol
SCID-5-CV: Structured Clinical Interview for DSM-5 Disorders—Clinician version
TDI: threshold-discrimination-identification

## References

[1] Hoffman HJ, Ishii EK, MacTurk RH (1998). Age-related changes in the prevalence of smell/taste problems among the United States adult population. Results of the 1994 disability supplement to the National Health Interview Survey (NHIS). Ann N Y Acad Sci.

[2] Gopinath B, Sue CM, Kifley A, Mitchell P (2012). The association between olfactory impairment and total mortality in older adults. J Gerontol A Biol Sci Med Sci.

[3] Schiffman SS, Graham BG (2000). Taste and smell perception affect appetite and immunity in the elderly. Eur J Clin Nutr.

[4] Doty RL (2017). Olfactory dysfunction in neurodegenerative diseases: is there a common pathological substrate?. Lancet Neurol.

[5] Serby M, Larson P, Kalkstein D (1991). The nature and course of olfactory deficits in Alzheimer's disease. Am J Psychiatry.

[6] Park JW, Kwon DY, Choi JH, Park MH, Yoon HK (2018). Olfactory dysfunctions in drug-naive Parkinson's disease with mild cognitive impairment. Parkinsonism Relat Disord.

[7] Cho JH, Jeong YS, Lee YJ, Hong SC, Yoon JH, Kim JK (2009). The Korean version of the Sniffin’ stick (KVSS) test and its validity in comparison with the cross-cultural smell identification test (CC-SIT). Auris Nasus Larynx.

[8] Oh SR, Chang MY, Kang H, Kim KS, Mun SK, Lee SY (2019). First recognized n-butanol concentration may be simple and useful index for assessing olfactory dysfunction in geriatric patients. Aging Clin Exp Res.

[9] Devanand DP, Michaels-Marston KS, Liu X, Pelton GH, Padilla M, Marder K (2000). Olfactory deficits in patients with mild cognitive impairment predict Alzheimer's disease at follow-up. Am J Psychiatry.

[10] Ha JG, Kim J, Nam JS, Park JJ, Cho HJ, Yoon JH (2019). Normative values for the YSK olfactory function test and optimization for the diagnostic cut-off. Proceedings of the 25th academic conference of otorhinolaryngology-head and neck surgery.

[11] Jin SY, Jeong HS, Lee JW, Kwon KR, Rha KS, Kim YM (2016). Effects of nutritional status and cognitive ability on olfactory function in geriatric patients. Auris Nasus Larynx.

[12] Doty RL, Wylie C, Potter M, Beston R, Cope B, Majam K (2019). Clinical validation of the olfactory detection threshold module of the Snap & Sniff ® olfactory test system. Int Forum Allergy Rhinol.

[13] American Psychiatric Association. Diagnostic and Statistical Manual of Mental Disorders (DSM-5®) Washington, DC: American Psychiatric Publishing; 2013.

[14] Lee JH, Lee KU, Lee DY, Kim KW, Jhoo JH, Kim JH (2002). Development of the Korean version of the consortium to establish a registry for Alzheimer’s disease assessment packet (CERAD-K): clinical and neuropsychological assessment batteries. J Gerontol B Psychol Sci Soc Sci.

[15] Shin MH, Lee YM, Park JM, Kang CJ, Lee BD, Moon E (2011). A combination of the Korean version of the mini-mental state examination and Korean dementia screening questionnaire is a good screening tool for dementia in the elderly. Psychiatry Investig.

[16] Lee DY, Lee KU, Lee JH, Kim KW, Jhoo JH, Kim SY (2004). A normative study of the CERAD neuropsychological assessment battery in the Korean elderly. J Int Neuropsychol Soc.

[17] Hedner M, Larsson M, Arnold N, Zucco GM, Hummel T. Cognitive factors in odor detection, odor discrimination, and odor identification tasks. J Clin Exp Neuropsychol. 2010;32:1062–7. 10.1080/13803391003683070 20437286

[18] Jiang RS, Kuo LT, Wu SH, Su MC, Liang KL. Validation of the applicability of the traditional Chinese version of the University of Pennsylvania Smell Identification Test in patients with chronic rhinosinusitis. Allergy Rhinol (Providence). 2014;5:28–35. 10.2500/ar.2014.5.0084 PMC4019742 25199144

[19] Shu CH, Yuan BC, Lin SH, Lin CZ (2007). Cross-cultural application of the “Sniffin’ sticks” odor identification test. Am J Rhinol.

[20] Schubert CR, Cruickshanks KJ, Fischer ME, Huang GH, Klein BE, Klein R (2012). Olfactory impairment in an adult population: the beaver dam offspring study. Chem Senses.

[21] Kobal G, Klimek L, Wolfensberger M, Gudziol H, Temmel A, Owen CM (2000). Multicenter investigation of 1,036 subjects using a standardized method for the assessment of olfactory function combining tests of odor identification, odor discrimination, and olfactory thresholds. Eur Arch Otorhinolaryngol.

