# Supplementary figures and images for: The sniffing bead system, an olfactory dysfunction screening tool for geriatric subjects: a cross-sectional study
Source: BMC Geriatr. 2021 Jan 14;21:54. doi: 10.1186/s12877-020-01871-7 (PMC7807818; doi:10.1186/s12877-020-01871-7)

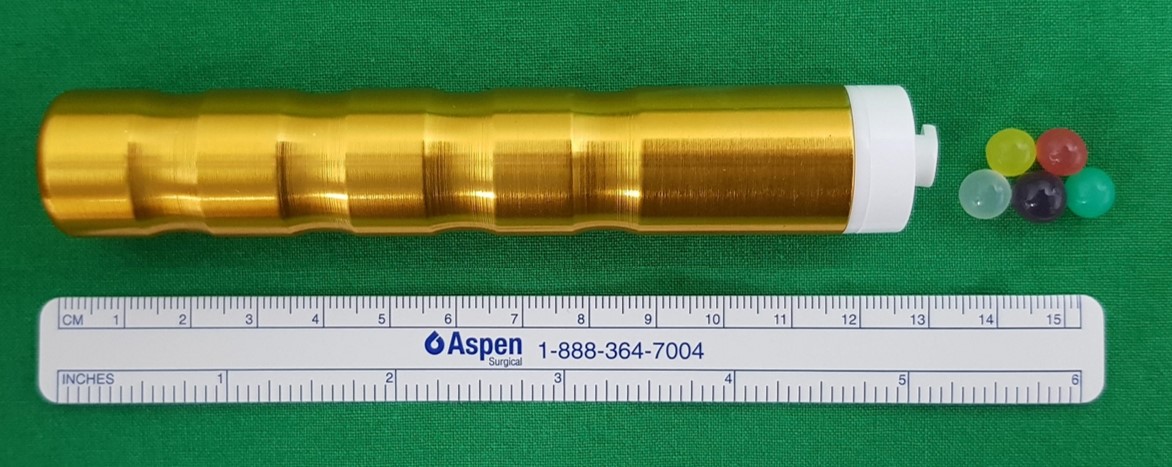

Supplement: Supplementary file 2 — Additional file 1: Figure S1. Picture of sniffing bead system. [file 12877_2020_1871_MOESM1_ESM.jpg]

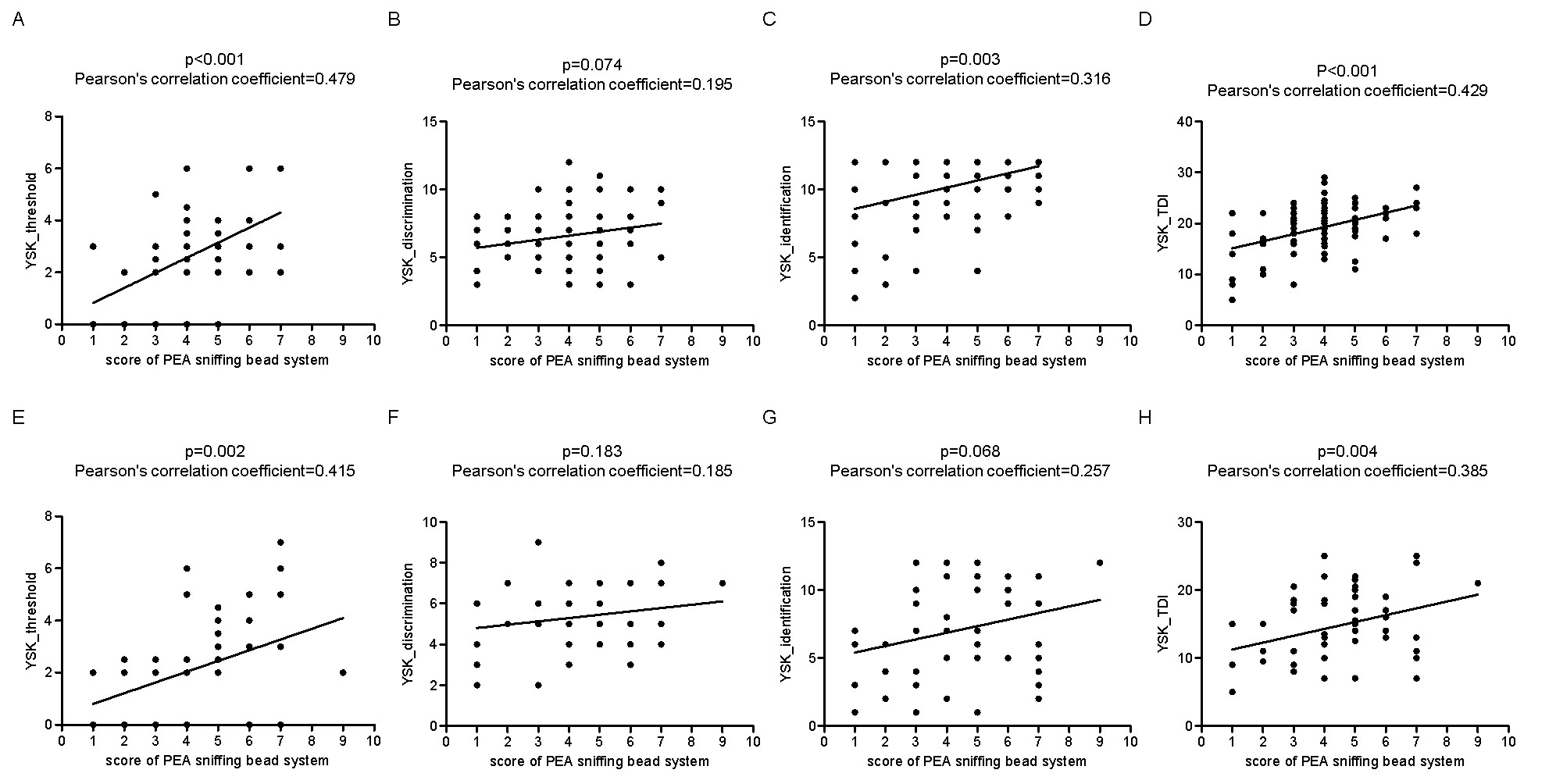

Supplement: Supplementary file 3 — Additional file 3: Figure S2. Correlation between each component of the olfactory function test (YSK) and the PEA sniffing bead system scores in subjects with normal cognitive function (A–D) and impaired cognitive function (E–H). [file 12877_2020_1871_MOESM3_ESM.jpg]

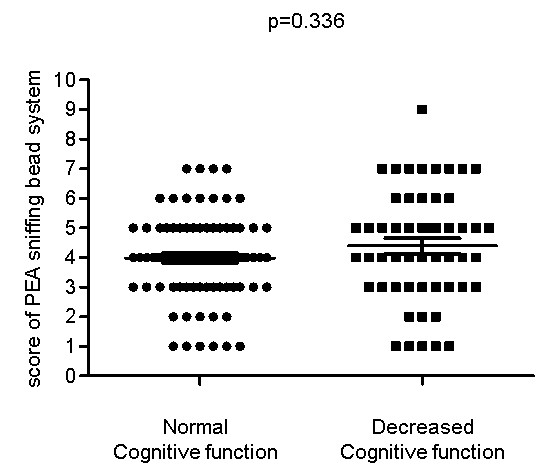

Supplement: Supplementary file 4 — Additional file 4: Figure S3. Comparison of the PEA sniffing bead system scores between subjects with normal and decreased cognitive function. [file 12877_2020_1871_MOESM4_ESM.jpg]
